# Standard Biological Parts Knowledgebase

**DOI:** 10.1371/journal.pone.0017005

**Published:** 2011-02-24

**Authors:** Michal Galdzicki, Cesar Rodriguez, Deepak Chandran, Herbert M. Sauro, John H. Gennari

**Affiliations:** 1 Biomedical & Health Informatics, University of Washington, Seattle, Washington, United States of America; 2 BIOFAB, University of California, Berkeley, California, United States of America; 3 Bioengineering, University of Washington, Seattle, Washington, United States of America; Kyushu Institute of Technology, Japan

## Abstract

We have created the Knowledgebase of Standard Biological Parts (SBPkb) as a publically accessible Semantic Web resource for synthetic biology (sbolstandard.org). The SBPkb allows researchers to query and retrieve standard biological parts for research and use in synthetic biology. Its initial version includes all of the information about parts stored in the Registry of Standard Biological Parts (partsregistry.org). SBPkb transforms this information so that it is computable, using our semantic framework for synthetic biology parts. This framework, known as SBOL-semantic, was built as part of the Synthetic Biology Open Language (SBOL), a project of the Synthetic Biology Data Exchange Group. SBOL-semantic represents commonly used synthetic biology entities, and its purpose is to improve the distribution and exchange of descriptions of biological parts. In this paper, we describe the data, our methods for transformation to SBPkb, and finally, we demonstrate the value of our knowledgebase with a set of sample queries. We use RDF technology and SPARQL queries to retrieve candidate “promoter” parts that are known to be both negatively and positively regulated. This method provides new web based data access to perform searches for parts that are not currently possible.

## Introduction

The engineering of new biological systems has begun to demonstrate the advantages of leveraging living cells as machines for the production of medicine [1], nutrients [2], biofuels [3], [4], and as biosensors [5], [6]. Driving the growth of these new technologies are advances in the approaches and tools used to control cellular processes [7], [8] and to construct synthetic DNA [9], [10]. Synthetic Biology offers the promise to address some of the world's most challenging problems [11].

To facilitate the process of development, synthetic biologists apply principles of engineering (i.e. standardization, abstraction, and decoupling) to specify the design, assembly, and validation of new biological systems [12]. In other engineering fields, such as mechanical, electrical, and computer engineering, these principles have led to the highly successful methods used today to build robust and complex products. The multiple scales, diversity, and dynamics inherent to biological systems and materials necessitate the use of computational methods to help manage this complexity. Synthetic biologists need software tools that support the engineering process of biological systems [13]. Several such software tools are currently in development and aim to aid the design of new systems by predicting their behavior, TinkerCell [14], BioNetCAD [15], SynBioSS [16], [17], and BioJADE [18] planning the assembly process [19], and validating the design GenoCAD [20], [21], [22], [23]. Such design tools require computational access to a library of parts, specifically the ability to query such a library.

The ability of synthetic biologists to manipulate the composition of DNA sequence should allow researchers to engineer cells with desired behavior. In particular, the modification of the basic elements of genetic regulatory networks, or “gene circuits” [24] is representative of a class of elementary behaviors [25] and can be thought of as modular [26]. Therefore, the abstraction of these segments of DNA as *biological parts* [27] for the purpose of engineering has been broadly adopted. The success of this approach is especially visible in the context of the International Genetically Engineered Machines (iGEM) competition (igem.org) [28], as evidenced by the growing number of biological parts in the Registry of Standard Biological Parts (partsregistry.org/cgi/partsdb/Statistics.cgi)[29]. This collection of parts, created by undergraduate students and independent synthetic biology laboratories is a ready source of components for engineering new biological systems.

Our research goal is to build a computationally accessible library of information about standard biological parts for synthetic biologists. We will design this library to support part re-use by leveraging the engineering principles of standardization, decoupling, and abstraction. If synthetic biologists had easy access to information about previously used parts, they could use this information to more efficiently design and plan for the assembly of new genetic devices. When already available components exist, and have been shown to work, their reuse would allow a synthetic biologist to focus on meeting design requirements, rather than re-creating prior work of others.

In this manuscript we present the Standard Biological Parts knowledge base (SBPkb), our initial version of a biological parts library that supports remote queries. This library builds on knowledge from the Registry of Standard Biological Parts (partsregistry.org), which we describe below. We adapted and transformed data from the registry into SBOL-semantic, that describes standard biological parts using RDF. Next, we demonstrate how the SBPkb can be queried using standard RDF technology (SPARQL queries) to retrieve parts that may be relevant to a synthetic biologist. We take as our use case queries about promoter parts. In our results section, we show (a) that such queries cannot be pragmatically answered with current technologies, and (b) that our approach allows researchers to carry out query refinement. For the latter, we show that our promoter query can iteratively be made more specific, so that the query results in smaller lists of parts, and where these parts are more well-matched to specific design criteria.

## Catalog of Parts: The Registry of Standard Biological Parts

The Registry of Standard Biological Parts (partsregistry.org) is a repository of biological parts for synthetic biology. The Registry is hosted at MIT and provides services to store and distribute plasmid DNA that conforms to certain specifications and descriptive information, i.e., a physical store and distribution point for biological parts. The Registry website is a publicly available source of information about those parts. The website is partially designed as a wiki, and therefore Registry users can edit its content directly. Registry staff also curate this information. From the point of view of a user it is organized from two main perspectives: one about individual part records and the second as a catalog or listings of various parts. The Registry also provides help and documentation sections, as well as user management features, such as groups and a user authentication system. Web pages describing individual part records provide detailed descriptive information about the DNA sequence, its design, and the availability of the part as physical DNA stored at the Registry. The second perspective is the Catalog which can be browsed to explore the contents and discover new parts. This section of the site is subdivided into categories ranging from listings of parts by their expected function (e.g. constitutive promoters) to listings of parts used in specific projects. For example, each iGEM team has a page of all parts created and used throughout the duration of the competition. While the Registry faces challenges maintaining integrity between the information and the DNA repository [29], it is a unique and rich resource for the synthetic biology community.

There are more than 13,444 part records within the Registry. This is the largest collection of publically available parts for synthetic biologists. In addition, like other fields within modern molecular biology, synthetic biology faces additional and rapid growth of this data. Efforts to standardize the characterization [30], [31] and composition [27], [32] of parts are gaining momentum in the synthetic biology community. There is now a need to standardize the electronic form of the knowledge about these parts. In addition to the Registry of Standard Biological Parts there are new notable software efforts addressing the need to manage information about biological parts. The Joint BioEnergy Institute Registry (JBEIr) provides a web based inventory platform as well as a graphical sequence annotator [33]. Clotho, a software framework for synthetic biology, offers a suite of tools for the design and management of new biological systems [34]. Furthermore, there are also efforts to store quantitative models that describe and predict functions of synthetic biology systems such as SynBIOSS [16], [17] and the Repository of Standard Virtual Parts [35], [36]. These systems, just like the design tools we mentioned earlier, would benefit greatly from computational access to the information contained in the Registry.

## Transformation of Parts Data to SBOL-Semantic

To describe common concepts used in synthetic biology, we implemented SBOL-semantic, an information model for synthetic biology, using the Web Ontology Language (OWL). The Synthetic Biology Open Language (SBOL) (sbolstandard.org) is a collaborative effort of the Synthetic Biology Data Exchange Group to develop standards and technologies to facilitate information exchange for synthetic biologists. SBOL-semantic is based on the rough consensus of core synthetic biology concepts and their relationships and represents the semantics of synthetic biology theory and practice. We used an open process for the evolution and standardization of data models according to a framework for how data models in synthetic biology should be published [37]. This new work builds on the Provisional BioBrick Language (PoBoL) [38].

We have built SBOL-semantic using OWL so as to be compliant with Semantic Web information technology standards that allow SBOL data records to be read, manipulated, and interpreted using generic tools such as Protégé [39], RDFlib [40] and Sesame [41]. These tools were used for management of SBOL model structure, to create a scheme for unique identification of elements, and to reference the Sequence Ontology [42], a third party ontology. The choice of W3C recommended technology was made on the premise that modeling knowledge in a computable, standardized, and community supported format will provide long term benefit for the synthetic biology community. (See also our discussion and future work sections.)

**Figure 1:**
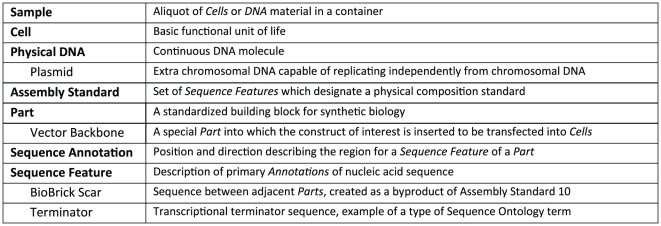
Top level *Class* (bold) and example *sub-class* (regular face) SBOL semantic terminology with a simplified definition for clarity.

**Figure 2:**
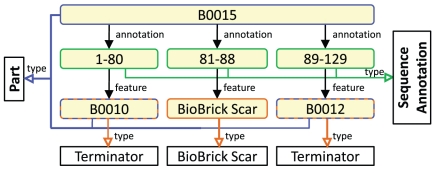
*Classes* (black rectangles) describe types (open faced arrows, colored by type) of *individual* data elements (yellow rounded rectangles) and the composition relationships between them (closed faced arrows).

The SBOL semantic structure is organized as a hierarchy of *classes* that refer to distinct categories of common information objects, such as Parts, Cells, Plasmids, and Sequence Features. The most general of the *classes* (Figure 1) constitute the core SBOL concepts. Instances of a *class* are *individual* data elements. Figure 2 shows the specific part known as BBa_B0015, a commonly used transcriptional terminator [29]. In this figure, the part has *annotations* that divide the part into segments such as BBa_B0010 that are themselves instances of the *Part* class. In our model, all such annotations are *properties* that capture relationship information between individuals. Data represented in this form can be conceptualized as a graph in which nodes are *individuals*, members of SBOL classes, and edges are the *properties* between them. Here we present results focused on *Parts* and the description of their nucleotide sequence, *Sequence Features*. The long term goal of SBOL is to represent information relevant to all levels of the engineering process in synthetic biology (Tissues, Cells, Plasmids, etc). Here, we demonstrate the open nature of the framework [37] by extending this class structure to support the needed concepts from the Registry.

To create the semantic knowledgebase for synthetic biology we used the information available from the Registry of Standard Biological Parts (partsregistry.org) to create an extension of the SBOL *class* structure. This extension uses SBOL-semantic in combination with the new terminology acquired from the Registry to describe biological parts. First, we extracted the Registry data and mapped its structure of tables, its relational schema, to SBOL-semantic. This mapping served as our translation table to transforming the Registry data of 13,444 part entries and the associated Sequence Features to OWL/RDF. Using a script, we converted 13,444 Registry part records with their associated Sequence Features from the Registry format to the SBOL semantic (OWL/RDF) form. Each Registry part record was also associated with the Registry's Sequence Feature table, a position based description of the nucleotide sequence (see Figure 2 for example sequence features such as a ‘terminator’). We then mapped the Registry Sequence Feature table to the SBOL Sequence Annotation and Feature Class structures and performed the analogous translation into OWL/RDF.

**Figure 3:**
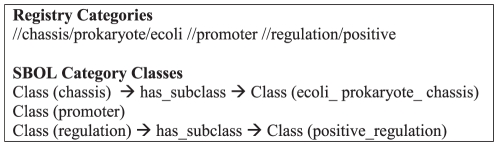
Example of Registry Categories to SBOL class structure conversion. These autogenerated classes are assigned to the partsregistry.org namespace to attribute them to the source and allow differentiation from SBOL-semantic classes, see the OWL implementation of SBOL-semantic File S1.

As part of the transformation of Registry data we used the categories attribute of the Registry *parts* table to provide a richer description of parts. The Registry includes a total of 346 categories organized as a hierarchy of 28 top level categories (e.g. chassis, classic, dna, function, plasmid, plasmidbackbone, primer, promoter, proteindomain, proteintag, rbs, regulation, ribosome, rnap, terminator, etc. For full listing see Supporting Information Table S1, which contains the list of terms extracted from the Registry data, and File S1., which contains the generated OWL encoded semi-structured controlled vocabulary used throughout this work). These categories are a rich vocabulary used to describe parts and constitute a controlled vocabulary, created and maintained by the Registry staff, while its use is enforced by the Registry website software application. The categories form the basis of organization for the Registry Catalog website. Thus, to provide a good structure for querying the Registry information, we needed to augment our core SBOL-semantic ontology with this terminology. To do so, we auto-generated a class structure within SBOL-semantic that mimics the registry category structure. For an example, see Figure 3. Finally, we loaded the SBOL-semantic data into a framework for querying RDF data, creating the Standard Biological Parts knowledgebase resource (SBPkb) (see Implementation and Availability for details). As we show in our results section, we can use these categories to directly query the SBPkb for specific features of parts.

The semi-structured controlled vocabulary resulting from this process does not fulfill many of the criteria of formal ontology design [43]. The structure created reflects the organization found in the Registry, and is not a proper class hierarchy. Our effort, directed towards SPARQL query information retrieval, translates the existing Registry information to a Semantic Web technology standard to enhance its potential for re-use. This utilitarian approach provides immediate benefit of data access and lays out the scope of the knowledge engineering challenges which face the synthetic biology community. Challenges of formally structuring information for future use in multiple applications are especially evident in large collections such as the user-driven and community-supported data source for our work, the Registry of Standard Biological Parts. However, the main contribution of this work is to provide a pragmatic solution for synthetic biology users, and establish the need for improvement of information resources in the field.

## Results

### The Case of the Promoter

To illustrate the functionality of SBPkb we describe a hypothetical case for its use to research the availability of promoters for a new design. We asked the knowledge base to answer the following question, “Which promoters can I use for a design?” Because “promoter” is a class in our controlled vocabulary, this is a straightforward SPARQL query to ask of our SBPkb (see query #1 in File S2), and it returns 538 parts that are annotated as promoters.

Although this query seems simple, we must compare the capabilities of SBPkb to current technology: How would one answer this question, with current technology, i.e., directly of the Parts Registry? Unfortunately, the only way to retrieve this set of parts is by manual browsing of web pages, and then manual compilation and analysis of the results listed on these web pages (also see the comparison section below). Additionally, SBPkb and SPARQL allow researchers to easily refine queries to both provide cleaner, more useful results, and to narrow the search to a more specific type of promoter. In this section, we describe how our initial query can be step-wise narrowed to a much more specific query that returns only six parts from our knowledgebase.

As a first step, we ask what information is associated with these parts—we carry out a SPARQL *describe* query (query #2 in File S2) that lists the complete set of properties associated with all promoters. This query would have a very long, large result, but we can sample only a few entries to explore the information space; Table 1 shows one sample entry from this query result. By looking at all available properties of a part, researchers may discover ways to narrow or improve their query. For example, an initial exploration may lead us to decide that the *status* property is important (we do not want any “deleted” parts), and that we only want parts that have DNA sequences listed. This refined query (query #3 in File S2 produces 529 parts (it eliminated seven “deleted” entries, and two without DNA sequences).

**Table 1:** Example result of a DESCRIBE SPARQL query for a selected single promoter part.

Subject	Predicate	Object
sbol:rQprqhqP5413	sbol:name	BBa_I746365
	rdf:type	sbol:ecoli_prokaryote_chassis
	rdf:type	sbol:sigma70_ecoli_prokaryote_rnap
	rdf:type	sbol:Part
	rdf:type	sbol:forward_direction
	rdf:type	sbol:promoter
	rdf:type	sbol:positive_regulation
	sbol:type	Regulatory
	sbol:shortDescription	PLL promoter from P4 phage
	sbol:longDescription	This is the PLL promoter taken from the P4 phage genome. It is an inducible promoter that is activated by a class of activators, including P2 ogr (I746350), PSP3 pag (I746351) and phiR73 delta (I746352). These different activators should cause different levels of activity of the PLL promoter.
	sbol:author	Stefan Milde
	sbol:status	Available
	sbol:id	9598
	sbol:owner_id	2122
	sbol:date	9/11/2007
	sbol:dnaSequence	cgctttattttgtgaatattttcagcagacgcaacaggggggatttgttcaggctgtcttacaatggctgtgtgttttttgttcatctccac

Trivially, we can also ask these sorts of “data cleaning” questions of the entire SPBkb. For example, we found that 12,152 of the 13,444 total part records have an associated DNA sequence and have not been marked for deletion (query #4 in File S2). Currently, many parts are larger in DNA sequence length than is financially prudent to directly synthesize, however not impossible using the latest methods [9]. Therefore, it is noteworthy that only 5,166 are marked as *Available* or as *Sent* to the Registry as clones (query #5 in File S2).

### Comparison with current capabilities

To validate our (cleaner) result of 529 promoter parts found via our SPARQL query and the SBPkb, we also attempted to answer this question by exhaustively browsing the Parts Registry. First, we dismissed an information retrieval approach that might use heuristic algorithms based on text searches of the word “promoter” within the Registry's web pages (e.g., a Google search). Although careful construction of good heuristics might lead to accurate results, a simple text search will result in many entries that mention “promoter” but are not themselves promoter parts.

Thus, we used an exhaustive manual method, systematically exploring all web pages in the ‘Promoter’ category of the Parts Registry Catalog. When information appears about parts, the Registry Catalog typically displays the information in a table. Therefore, whenever we encountered a page with parts labeled as a category promoters, we copied the corresponding table into a spreadsheet application (MS Excel). This exploration results in 42 separate web pages (many with several tables) and a total of 833 promoter parts. (This data was collected by MG on Aug 3, 2010 from partsregistry.org/Promoters/Catalog). Because the same part can be found on multiple web pages, the same part identifier can be copied onto the spreadsheet multiple times. We removed these duplicate entries using the Remove Duplicates Data Tool in Excel™ and obtained a unique list of 474 promoter entries. Finally, we noted that two of these lacked DNA sequence information, a requirement of our “cleaner” query.

The set of 472 entries that we found manually are all included in the set of 529 promoters returned by SBPkb. That is, there is no information “missed” by our knowledgebase. SBPkb also retrieved 57 additional entries that appear to be bona fide promoters, from a variety of subcategories. We attempted, but were unable to discover why these particular promoters were missing from our manual browsing of the web pages (see Table S2. for this list of 57 promoters).

It should be clear that exhaustive web page browsing is not a scalable approach to searching for a particular class of biological part. Indeed, the registry instead is a community-based, wiki-style collection of parts dedicated to capturing information about parts. Supporting such queries is a novel design consideration for a semantic web of data in synthetic biology. Query answering is a central design feature of the SPBkb, and as we demonstrate next, our initial query can be narrowed to return a much smaller set of parts, yet still maintain the ability to exhaustively search the knowledge base.

### Design query refinement

The process of query refinement, or improvement of the query, as a specification of information needs, involves exploration in order to discover information about a topic [44]. We again look through the results of (query #2 in File S2) to find additional criteria by which to search SBPkb. The query driven exploration process helped us discover the rich source of structured information derived from the Registry categories. Among the results of this query (Table 1), we found that the example promoter part belongs to the type or category, ‘sigma70_ecoli_prokaryote_rnap’. The categories, represented as OWL *classes* in SBOL semantic, provide the capability to refine queries for promoters. For example, to narrow the selection to only those promoters which are expected to work with the *Escherichia coli* RNAP σ^70^ holoenzyme (Eσ^70^) and therefore to have an expected peak efficiency at the exponential growth phase [45]. This query (query #6 in File S2) results in 367 “Eσ^70^” promoters, a subset of the 529 promoters found in our initial query. This list of 367 are the most likely candidates to use for common synthetic biology experiments in *E.coli* for which measurements are taken at mid-exponential phase. The capability of retrieving specific parts from the thousands of entries within SBPkb by selection criteria such as the *class* structure of biological system contexts will allow synthetic biologists to find parts relevant to their design.

**Figure 4:**
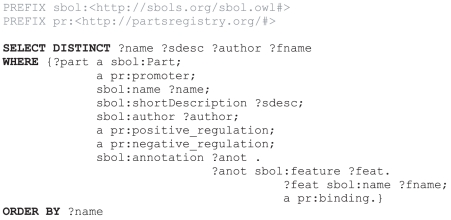
SPARQL query of SBPkb for dual-regulated promoter parts and their descriptions.

Not only were we able to retrieve promoter parts based on specific factors (σ), but available to us as selection criteria were also Registry categories which specify the expected mode of regulation. For example, during the design of a new genetic Barkai-Leibler oscillator [46], [47] the synthetic biologist may want to find all pre-existing promoters that can be both ‘positively regulated’ AND ‘negatively regulated’, i.e., dual-regulated promoters (query #7 in File S2). Our query returned just 36 unique promoter parts meeting these criteria (note that this query result is not necessarily a subset of the 367 “Eσ^70^” promoters). The Barkai-Leibler oscillator relies heavily on the availability of such dual-regulated promoters, therefore having knowledge of all dual-regulated promoters available in the Registry is highly advantageous to the synthetic biologist. Since a sufficient number of dual-regulated promoters are available, the search can be further limited to promoters for known specific inducers and repressors that are appropriate for the new design. The SBPkb includes information from the Registry Features table, therefore, for our final refinement, we further restricted our query to return promoters that have sequence annotations of known transcription factor binding sites, i.e., operator sites. This example query (Figure 4) returns just six parts and their known binding sites (Table 2). A selection of these six candidates provides a list small enough that each one can be examined in greater detail for relevance to a specific design.

**Table 2:** SBPkb promoter parts that can be both positively and negatively regulated with operator site sequence features.

Name	Short description	Author	Feature	Feature	Feature	Feature
BBa_I12036	Modified lamdba Prm promoter	Hans	OR1 lambda	OR2 lambda	OR1 434	OR2 434
BBa_I12006	Modified lamdba Prm promoter	mcnamara	OR1 lambda	OR2 lambda	OR1 434	
BBa_I12040	Modified lambda P(RM) promoter	ryhsiao	OR1 lambda	OR2 lambda	OR1 434	OR2 434
BBa_I14015	P(Las) TetO	Vijayan, V., Hsu, A., Fomundam, L.	TetR			
BBa_I14016	P(Las) CIO	Vijayan, V., Hsu, A., Fomundam, L.	CI lambda O1			
BBa_I1051	Lux cassette right promoter	Mahajan, V.S., Marinescu, V.D., Chow, B., Wissner-Gross, A.D., Carr, P.	cI (OR1)	LuxR/HSL		

During planning stages of a new synthetic biology research project investigation of prior work is an important phase of forming a new design. This process involves the exploration of available information resources for the purpose of discovery of candidate components to leverage in such a design. The SPARQL *describe* query in SBPkb can help identify information types or classes, such as Registry categories and data fields that hold information management, engineering, or biologically relevant information. These facts, or descriptions of parts, can then be used to search across the entire information collection to identify parts relevant to a particular design specification or criteria. This ability to quickly identify specific parts that match design criteria provides a method that enables fast and thorough exploration of prior work.

### Implementation and Availability

To construct SBOL semantic we used Protégé 4.0.133 (protege.stanford.edu) and used a RDFlib (rdflib.net), a python library to perform programmatic additions of class terms and individuals during the data import process. We obtained the Standard Biological Parts Registry data from (partsregistry.org/Registry_API) on April 6, 2010. The downloaded information was provided in the form of two MySQL tables formatted as XML, a table of parts and a table of Sequence Features. These were converted into a text based delimited format to serve as input for SBPkb. We created python import scripts to parse the input tables from the Registry and libSBOL, a python library, to aid population of SBOL structures to generate the RDF/XML form of the data for SBPkb (synbiolib.sourceforge.net).

We have made the SBPkb data accessible via SPARQL a W3C recommended query language for RDF queries, with remote access (through a RESTful HTTP interface) provided using the Sesame 2.3.1 (openrdf.org) software. The SBPkb (sbpkb.sbolstandard.org) as a SPARQL accessible knowledge base is a publically available Semantic Web computational resource for the synthetic biology community.

## Discussion

To effectively build new systems from prior work and best practices, synthetic biologists developed an initial framework and standards for the description of engineered biological devices [30], [31]. The common approach of storing data about biological parts in a spreadsheet is convenient for a small laboratory. Our experience in synthetic biology research suggests that sharing such information between collaborating laboratories requires a significant coordination effort. Furthermore, *ad hoc* organization of part description information is too ambiguous to establish an efficient engineering pipeline for synthetic biology. The process of engineering synthetic biological systems relies on specialized software tools to: model systems, aid design, and plan assembly. For software to help researchers make appropriate design decisions, biological parts must be described using an unambiguous language, such as SBOL-semantic. To reconcile the need for engineering with base pair precision with the inherent complexity of biological system dynamics at multiple scales, there is a need for software tools to have the ability to exchange information about the entire spectrum of the domain of synthetic biology. Working towards the goal of defining an unambiguous computational language for synthetic biology, we have created Standard Biological Parts Knowledgebase (SBPkb). This public resource uses the Synthetic Biology Open Language semantics (SBOL-semantic) as its organizing structure and demonstrates its use for information retrieval.

Current methods for finding previously described biological parts are insufficient to realize new synthetic biology designs with increased sophistication. To create such integrated systems from parts and modules synthetic biologists must overcome significant challenges posed by the uncertainty and complexity of biology [48]. Synthetic biologists need to be able to draw on large numbers of examples of prior work to learn from the successes and failures of previous efforts. We have populated the SBPkb with the thirteen thousand parts from the Registry of Standard Biological Parts, and we have made it available for public use. Purnick & Weiss [48] reported that the most complex system built up to that time, as measured by the number of regulatory regions within a design, was six. Automatically searching the SBPkb, for existing candidate parts, will increase the number of part options to consider in designs. This ability, to quickly query part information from the large repository of knowledge provided by the Registry, removes one significant barrier in the exploration of prior work.

The ability to query SBPkb using a remote query protocol can serve to extend the capabilities of computational tools which support design work. Software designed to help synthetic biologists to plan designs can greatly benefit from a computationally accessible search interface. Information retrieved from SBPkb by SPARQL is returned as SBOL-semantic RDF/XML therefore can easily interpreted by the receiving application. For example, TinkerCell [14], [49], a computer aided design application, could use SBPkb queries to fulfill designs based on combinations of specific requirements. We demonstrated one such hypothetical query for promoter parts controlled by dual modes of regulation. TinkerCell, and other design tools, could take advantage of query results to suggest these candidate parts to a user who is building a new Barkai-Leibler oscillator. The use of query refinement as a method for specifying design requirements would be an important methodological development towards automating the design to production pathway in synthetic biology.

SBOL-semantic is based on the robust principles and technology developed by the Semantic Web research program. The utility of the approach we described provides information retrieval services via a standard query language, SPARQL. However, we look forward to building on the foundation established by the SBOL-semantic framework to support additional capabilities, specifically to take advantage of reasoning services for ontologies formalized in OWL. Semantic Web inference engines, such as Pellet [50], Hermit [51], and Fact++ [52] perform consistency checking and classification/realization. These tools validate and generate new inferences about a set of axioms based on logical constraints and restrictions defined in OWL. Therefore, to develop significant improvements to SBOL-semantic, the terms from the controlled vocabulary provided by the Registry will have to conform with ontology design best practices [43] and be defined using OWL-DL class restrictions. Therefore, to impart these capabilities we plan to formalize SBOL-semantic class definitions to make SBOL-semantic into an authoritative ontology for synthetic biology.

To aid in the design of transcriptional devices, we will extend SBOL-semantic in order to describe rules for how components can be combined together [22] and regulated. For example, to specify the interaction between transcriptional regulatory proteins and their cognate sequences, we will use simplified representation of functional relationships. Towards this goal we plan to leverage related work such as the BioPAX effort (biopax.org) [53], [54] to specify the potential role of a promoter and factor pair, not the mechanism by which it occurs. A qualitative relationship between promoter parts and regulatory proteins will allow us to query and infer intended and unintended interactions. (The ability to carry out such inferences will require the use of a Semantic Web inference system such as Pellet.) For example, an instance of the promoter pLuxR (BBa_R0062) can be annotated as having an activating role on downstream expression in presence of LuxR protein and 3-oxo-hexanoyl-HSL (3OC_6_HSL). Such a representation of gene regulation information is limited, but forms a framework for regulatory element information retrieval. In general, we aim to expand SBOL-semantic so that it can support consistency checking of designs as a way to do initial validation of a design and to help identify possible design problems early in the engineering process.

### Summary and Future Directions

Due to the amount of detail inherent in any biological system and the distributed nature of scientific research, a semantic-web based solution for organizing synthetic biology data is the suitable choice. The SBOL-semantic framework described in this work can be used to unambiguously describe, remotely query, and therefore electronically retrieve information about biological parts. In the ideal scenario, researchers would use front-end software applications for submitting and retrieving parts from the SBPkb. SBOL-semantic plug-ins for TinkerCell and Clotho are already being planned to allow those software applications to export and import parts made available through SBPkb. Embedding SBPkb query utilities in the user friendly graphical interfaces of software will help us bring these capabilities into the workflow of active synthetic biologists.

Synthetic biology research is highly distributed. In the future we envision, not just a single library, but a network of libraries. Such part libraries may range from those that contain predominately parts described in peer reviewed publications, or be a collection of parts professionally fabricated by organizations such as the International Open Facility Advancing Biotechnology (BIOFAB). As long as all these libraries are compatible with SBOL-semantic, then researchers can retrieve parts from any selection of these libraries. The SBPkb is the first node in a framework for a semantic web of distributed knowledge in synthetic biology. This vision is a small scale synthetic biology application of the Semantic Web.

In the validation portion of this work we demonstrated that searching for part information using a manual process is not a scalable or pragmatic method. Searching the web pages requires manual compilation and curation for each information query; such methods are not scalable in the face of the continually growing number of available biological parts. Using SBOL-semantic to describe synthetic biology concepts not only allows electronic retrieval, but offers the ability to select specifically defined subsets of parts.

We plan to improve and extend SBOL-semantic in the near future. Our goal is to re-engineer SBOL-semantic into an ontology which supports the forward engineering practice of synthetic biologists. In particular, we aim to include enough information to support consistency checking and design coherence, as described in the discussion section. By automating reasoning, using the semantic definitions of biological components, we aim to provide improved design automation functionality for CAD software, such as TinkerCell. More broadly, we expect to leverage the ability of the OWL language to capture rich semantics, and to support ‘intelligent’ information retrieval and reasoning capabilities as envisioned by the Semantic Web. This further integration of SBOL-semantic with software will help encourage re-use of previously described components, a best practice of synthetic biology.

Additionally, we hope to work with the developers of other computational tools for synthetic biologists which could benefit from computational access to a large repository of knowledge about standard parts. SBOL is an open language. The success of the language, as well as that of the broader effort to standardize electronic information exchange in synthetic biology, depends on the active involvement of the interested community. We therefore extend an invitation to all interested readers to participate in the Synthetic Biology Data Exchange Group (sbolstandard.org and the discussion forum groups.google.com/group/synbiodex).

Reuse of components in synthetic biology research is one key way in which biologists can more easily engineer and construct new systems with increased complexity. The SBOL framework allows us to capture the semantics of richly-structured descriptions and to incorporate new information needed for design in synthetic biology. Automation of design promises to make building biological machines more efficient. Finding parts that meet the specifications of designs is a critical aspect of automation of the engineering process. Leveraging Semantic Web tools (such as SPARQL) to perform information retrieval can fulfill this need and offer additional benefits such as consistency checking and classification through automated inference. Adopting these capabilities to biological system design should allow engineers to use previously created solutions and apply them to solve novel problems.

## Supporting Information

File S1 SBOL-semantic OWL file which contains the semi structured controlled vocabulary used to describe standard biological parts in the SBPkb, created August 24, 2010.
(TAR)

File S2 Text file containing SPARQL queries used to retrieve standard biological parts from SBPkb.
(DOC)

Table S1 List of Part Registry Categories, attributes obtained from the source database table.
(XLSX)

Table S2 Promoter parts discovered using SBPkb query, but not found during the manual browsing portion of our work. The descriptions of the 57 additional entries, such as the status and categories are shown in the table and do not reveal a pattern which would explain their exclusion from the Catalog portion of the Parts Registry website.
(XLSX)
