# Supplementary material for: Standard Biological Parts Knowledgebase
Source: PLoS One. 2011 Feb 24;6(2):e17005. doi: 10.1371/journal.pone.0017005 (PMC3044748; doi:10.1371/journal.pone.0017005)
Supplement: File S2 — Text file containing SPARQL queries used to retrieve standard biological parts from SBPkb. (DOC) [file pone.0017005.s002.doc]

## #File S1 Supplementary Information Galdzicki, et al. Standard Biological Parts Knowledgebase

## # SBPkb SPARQL queries used.

## #The SPARQL endpoint, where these queries can be executed, is publicly accessible:

## # http://sbpkb.sbolstandard.org

**# Query 1**

**#Select all 538 promoter Parts**

PREFIX sbol:<http://sbols.org/sbol.owl#>

PREFIX pr:<http://partsregistry.org/#>

SELECT DISTINCT ?name

WHERE {

?part a sbol:Part;

a pr:promoter;

sbol:name ?name.

}

**# Query 2**

**#DESCRIBE promoter Parts**

PREFIX sbol:<http://sbols.org/sbol.owl#>

PREFIX pr:<http://partsregistry.org/#>

DESCRIBE ?part

WHERE {

?part a sbol:Part;

a pr:promoter;

sbol:name ?name.

} ORDER BY ?part

LIMIT 50 #very long results

**# Query 3**

**#Find all 529 promoters**

PREFIX sbol:<http://sbols.org/sbol.owl#>

PREFIX pr:<http://partsregistry.org/#>

SELECT DISTINCT ?name

WHERE {

?part a ?cl;

sbol:status ?st;

sbol:name ?name;

sbol:dnaSequence ?seq;

FILTER (?cl = pr:promoter && ?st !='Deleted')

}

**# Query 4**

#**Find all 12152 Parts with DNA sequence that are not Deleted**

PREFIX sbol:<http://sbols.org/sbol.owl#>

SELECT DISTINCT ?name

WHERE {

?part a sbol:Part;

sbol:name ?name;

sbol:status ?st;

sbol:dnaSequence ?seq

FILTER (?st !='Deleted')}

# **Query 5**

#**Find all 5166 Parts Available or Sent with Sequence**

PREFIX sbol:<http://sbols.org/sbol.owl#>

SELECT DISTINCT ?name

WHERE {

?part a sbol:Part;

sbol:name ?name;

sbol:dnaSequence ?seq;

sbol:status ?st;

FILTER (?st ='Available' || ?st='Sent')}

**# Query 6**

**#Find all 367 promoters known to work in E.coli with s70 holenzyme (Es70) aka sD**

PREFIX sbol:<http://sbols.org/sbol.owl#>

PREFIX pr:<http://partsregistry.org/#>

SELECT DISTINCT ?name

WHERE {

?part a sbol:Part;

sbol:status ?st;

sbol:name ?name;

sbol:dnaSequence ?seq;

a pr:promoter;

a ?cl.

FILTER (?cl =pr:sigma70_ecoli_prokaryote_rnap

&& ?st !='Deleted')}

**# Query 7**

**#Select + and - regulated promoters**

PREFIX sbol:<http://sbols.org/sbol.owl#>

PREFIX pr:<http://partsregistry.org/#>

SELECT DISTINCT ?name ?sdesc ?author ?fname

WHERE {?part a sbol:Part;

a pr:promoter;

sbol:name ?name;

sbol:shortDescription ?sdesc;

sbol:author ?author;

a pr:positive_regulation;

a pr:negative_regulation.

OPTIONAL{?part sbol:annotation ?anot.

?anot sbol:feature ?feat.

?feat sbol:name ?fname;

a pr:binding.}

}ORDER BY ?name

**#36 parts, in 45 rows**

**# Other Queries used to generate results but not cited explicitly in article #**

**#Figure 3 Query**

**#Select +/- regulated promoters**

PREFIX sbol:<http://sbols.org/sbol.owl#>

PREFIX pr:<http://partsregistry.org/#>

SELECT ?name ?sdesc ?author ?fname

WHERE {?part a sbol:Part;

a pr:promoter;

sbol:name ?name;

sbol:shortDescription ?sdesc;

sbol:author ?author;

a pr:positive_regulation;

a pr:negative_regulation;

sbol:annotation ?anot .

?anot sbol:feature ?feat.

?feat sbol:name ?fname;

a pr:binding.

}ORDER BY ?name

**#Find All 13444 Parts**

PREFIX rdfs:<http://www.w3.org/2000/01/rdf-schema#>

PREFIX rdf:<http://www.w3.org/1999/02/22-rdf-syntax-ns#>

PREFIX sbol:<http://sbols.org/sbol.owl#>

SELECT DISTINCT ?part

WHERE {

?part rdf:type sbol:Part;

}

**#Find all Sequence Features**

PREFIX rdfs:<http://www.w3.org/2000/01/rdf-schema#>

PREFIX rdf:<http://www.w3.org/1999/02/22-rdf-syntax-ns#>

PREFIX sbol:<http://sbols.org/sbol.owl#>

SELECT DISTINCT ?feat

WHERE {

?feat rdf:type sbol:SequenceFeature

}

**#Find all Sequence Annotations**

PREFIX rdfs:<http://www.w3.org/2000/01/rdf-schema#>

PREFIX rdf:<http://www.w3.org/1999/02/22-rdf-syntax-ns#>

PREFIX sbol:<http://sbols.org/sbol.owl#>

SELECT DISTINCT ?anot

WHERE {

?anot rdf:type sbol:SequenceAnnotation

}
